# Effect of Shengyang Yiwei Decoction combined with selective serotonin reuptake inhibitor antidepressants on HAMD-17 score and somatic symptoms in patients with depression

**DOI:** 10.12669/pjms.41.1.9835

**Published:** 2025-01

**Authors:** Lin Li, Aihua Wan, Mingkun Zhao, Liyan Yu, Yanchao Han, Kun Mi

**Affiliations:** 1 Lin Li, Integrated Traditional Chinese and Western Medicine Department, Hebei Provincial Mental Health Center, Baoding 071000, Hebei, China; 2 Aihua Wan, Integrated Traditional Chinese and Western Medicine Department, Hebei Provincial Mental Health Center, Baoding 071000, Hebei, China; 3 Mingkun Zhao, Integrated Traditional Chinese and Western Medicine Department, Hebei Provincial Mental Health Center, Baoding 071000, Hebei, China; 4 Liyan Yum, Integrated Traditional Chinese and Western Medicine Department, Hebei Provincial Mental Health Center, Baoding 071000, Hebei, China; 5 Yanchao Han, Integrated Traditional Chinese and Western Medicine Department, Hebei Provincial Mental Health Center, Baoding 071000, Hebei, China; 6 Kun Mi, Integrated Traditional Chinese and Western Medicine Department, Hebei Provincial Mental Health Center, Baoding 071000, Hebei, China

**Keywords:** Shengyang Yiwei Decoction, Selective Serotonin Reuptake Inhibitor, Depression, HAND-17, Somatic symptoms

## Abstract

**Objective:**

To investigate the efficacy of Shengyang Yiwei Decoction (SYD) combined with selective serotonin reuptake inhibitor(SSRI) antidepressants on the total score and scores of factors of the Hamilton Rating Scale for Depression(HAMD-17) and somatic symptoms in patients with depression.

**Methods:**

This was a retrospective study. One hundred and twenty patients with depression were treated in Hebei Provincial Mental Health Center between December 2020 and May 2022 and randomly divided into the experimental group and the control group, with 60 patients in each group. Patients in the control group were treated with selective serotonin reuptake inhibitor (SSRI) antidepressants and those in the experimental group were treated with modified SYD in addition to SSRIs for eight weeks. Explored the efficacy and adverse drug reactions of the two treatment options.

**Results:**

After eight weeks of treatment, there was no statistically significant difference in the total efficacy between the two groups(p>0.05); the total score of HAMD-17 and scores of factors except body weight were decreased in the experimental group compared with those in the control group, and the differences were statistically significant(p<0.05). The SSI score of the experimental group was decreased compared with that of the control group, and the difference was statistically significant(p<0.05).

**Conclusion:**

SYD combined with SSRI antidepressants in the treatment of depression can reduce the total score and the scores of factors of HAMD-17 without increasing the incidence of adverse drug reactions. This combination can be used as an integrated traditional Chinese and Western medicine treatment for depression.

## INTRODUCTION

Depression is a common mental disease with three main clinical manifestations. The core symptoms include low mood and decreased interest; the psychological symptoms include anxiety and cognitive impairment; and the somatic symptoms include pain, functional dyspepsia, irritable bowel symptoms, and sleep disorders.^1^ The incidence of depression is rising rapidly, and it is estimated that the disease burden of depression may rise to the first place in China by 2030.^2^ Although various antidepressants are currently used in clinical practice, only 60% patients respond to the treatment.^3^

A study on initial treatment with antidepressants in other countries showed that the remission rate of depressive symptoms after treatment is less than 50%^4^, and one of the reasons for the poor efficacy is the poor relief of somatic symptoms. The incidence of somatic symptoms in patients with depression can be higher than 70%, and even up to 100% as found in a survey done in China.^5^ The clinical manifestations of depression can be classified into the category of depression syndrome in traditional Chinese medicine (TCM) theory, and its pathogenesis involves dysfunction of organs such as heart, liver, spleen, lung, and kidney, as well as pathological factors such as qi, fire, phlegm, and blood stasis.^6^ Particularly, this disease is closely related to spleen and stomach functions.

Most somatic symptoms also involve spleen and stomach dysfunction. Shengyang Yiwei Decoction (SYD), a classic prescription in “Treatise on the spleen and stomach”, is specifically designed for the pathogenesis of spleen deficiency and damp heat, and primarily intended to treat symptoms such as listlessness and painful joints, dry mouth and tongue, tasteless, irregular bowel movements, frequent urination, lack of appetite, and inability to digest food with concurrent pulmonary diseases, chills, sadness, and disharmony in the face. On this basis, SYD combined with selective serotonin reuptake inhibitor (SSRI) antidepressants were selected for the treatment of depression in the present study to investigate their effects on the HAMD-17 score and somatic symptoms of patients, providing an evidence-based basis for the clinical treatment of depression and offering new ideas and methods for the improvement of depressive symptoms.

## METHODS

This was a retrospective study. One hundred and twenty patients with depression treated in Hebei Provincial Mental Health Center between December 2020 and May 2022 were selected and divided into the control group(n=60) and the experimental group(n=60) using a random number table. There were 27 males and 33 females in the control group, with an average age of 47.2±16.7 years old (range 18-70 years), and 28 males and 32 females in the experimental group, with an average age of 46.7±15.6 years old (range 18-70 years).

### Ethics Approval:

This study was approved by the Medical Ethics Committee of Hebei Provincial Mental Health Center (No.:2020266; date: September 01, 2019). All patients enrolled in the study signed informed consent forms.

### Inclusion criteria:

- Met the diagnostic criteria for depressive episode (single episode or recurrent) in the Mental and Behavioral Disorders in the ICD-11.^7^
- With an age of 18 to 70 years old, males or females.
- Treated with SSRI antidepressant monotherapy.
- With a HAMD-17 score of >17 points.
- With an education level higher than junior high school, and with normal cognitive, listening, and language communication abilities.
- Who signed informed consent to participate in and cooperate with the study.

### Exclusion criteria:

- With mental disorders such as bipolar disorder, mental retardation, and personality disorders.
- With comorbidities such as severe physical diseases.
- Undergoing modified electro-convulsive therapy (MECT).
- Who had a significant risk of suicide or were assessed by the investigator to have a significant risk of suicide.

Patients in the control group were conventionally treated with SSRI antidepressants for eight weeks, commonly used drugs include sertraline, fluoxetine, fluvoxamine, paroxetine, citalopram, etc., the dose was adjusted by the doctor in charge according to the conditions of the patients. Benzodiazepines, zolpidem, zopiclone and other drugs could be used for patients with poor sleep. Medications for comorbidities were allowed to continue during the study.

In addition to the treatment of the control group, patients in the experimental group were treated with modified SYD using the TCM formula granules made by Yiling Pharmaceutical. Formula compositions included Astragalus membranaceus 30 g, Pinellia ternata 8 g, Panax ginseng 3 g, prepared licorice 5 g, Saposhnikovia divaricate 5 g, Radix Paeoniae Alba 10 g, radix angelicae tuhuo 3 g, notopterygium root 3 g, pericarpium citri reticulatae 5 g, Poria cocos 10 g, radix bupleuri 5 g, Atractylodes macrocephala10 g, and Coptis chinensis 6 g. One dose were administered daily, and the daily dose was divided into two equal parts, with one part taken in the morning and one in the evening. The treatment continued for eight weeks, and follow-up for six months.

### Outcome measures:

Clinical efficacy evaluation: The HAMD-17 scores were evaluated before treatment and after eight weeks of treatment. Efficacy was evaluated as markedly effective if the total score was reduced by ≥75%, effective if the total score was reduced by 50%-74%, and ineffective if the total score was reduced by ≤49%.^8^ Total efficacy was calculated as the sum of the markedly effective rate and efficacy.

Assessment of each factor of HAMD-17 scale and somatic symptoms: The severity of depression and somatic symptoms of patients were assessed using the HAMD-17 scale and SSI one day before treatment and after eight weeks of treatment. HAMD-17 is a clinician-administered tool with 17 items in five factors. Among these factors, the anxiety/somatic factor included six items, including psychic anxiety, somatic anxiety, gastrointestinal symptoms, hypochondriasis, general symptoms, and insight; the body weight factor included weight loss; the cognitive impairment factor included three items: feeling of guilt, suicide, and agitation; the retardation factor included four items, i.e., depressed mood, work and interests, retardation, and sexual symptoms; and sleep disorder factor included difficulty in falling asleep, lack of deep sleep, and wakening in early hours. The SSI is a self-rating scale with 28 items and a total score of 140 points. A score of >56 points indicated the presence of moderate to severe somatic symptoms. All the questionnaire was given to the participants for investigation, and then collected in about 5 minutes.

### Safety evaluation:

Adverse drug reactions were evaluated using the Treatment Emergent Symptom Scale (TESS) one day before treatment and after eight weeks of treatment. TESS included 36 items, and the presence of an adverse reaction was considered if a score of ≥2 points was obtained for each item.

### Statistical Analysis:

SPSS 22.0 was used for data analysis. The confidence interval is 95%, and the total effective rates were enumeration data and presented as percentage, and χ^2^ test was used for comparison between groups. Measurement data such as HAMD-17 and SSI scores were presented as mean ± standard deviation (x̄±*S*) for those with normal distribution and equal variance, and paired sample t-test was used for comparison within groups and independent t or analysis of variance for comparison between groups. Data with non-normal distribution or unequal variance and ranked data were analyzed using non-parametric tests. Differences with a p value of <0.05 were considered statistically significant.

## RESULTS

A total of 120 patients who met the inclusion criteria were selected. Seven patients dropped out or were excluded from the control group, including three patients who withdrew their informed consent Forms, two with poor treatment compliance, one with out-of-window visit, and one with planned pregnancy, with a dropout rate of 11.7%. Five patients dropped out or were excluded from the experimental group, including one patient who withdrew the informed consent Form, one with fluctuated condition that required MECT treatment, one with out-of-window visit, one with poor treatment compliance, and one with the risk of mania switch, with a dropout rate of 8.3%. Patients dropped out and excluded were included in the ITT analysis.

There were no statistically significant differences between the two groups in terms of general data and the dosage of antidepressants (converted to fluoxetine equivalent)(P>0.05)(Table-I). No significant difference in the total efficacy was observed between the two groups after eight weeks of treatment (P>0.05) (Table-II). The total score and scores of factors except body weight factor were decreased in the experimental group compared with those in the control group, and the differences were statistically significant (Ρ<0.05) (Table-III).

**Table-I:** Comparison of general data between the two groups.

Items	The control group (n=60)	The experimental group (n=60)	t/χ^2^	Ρ
Sex, n (%)			0.03	0.86
M	27(45.0)	28(46.7)		
F	33(55.0)	32(53.3)		
Age, years(`*χ*±*s*)	47.2±16.7	46.7±15.6	-0.17	0.87
Use of antidepressants, n (%)			2.36	0.50
Sertraline	11(18.3)	9(15.0)		
Escitalopram	29(48.3)	29(48.3)		
Paroxetine	9(15.0)	15(25.0)		
Citalopram	4(6.7)	2(3.3)		
Fluoxetine equivalent(mg,` *χ*±*S*)	36.0±12.4	39.7±11.0	-1.73	0.08

**Table-II:** Comparison of clinical efficacy between the two groups.

Groups	n	Markedly effective, n	Effective, n	Ineffective, n	Total effective rate, n(%)
The control group	60	13	25	22	38(63.3)
The experimental group	60	23	24	13	47(78.3)
*c* ^2^					3.267
*P*					0.071

**Table-III:** Comparison of the total score and scores of factors of HAMD-17 between the two groups.

Groups	n	Total score		Anxiety/somatic factor		Body weight factor		Cognitive impairment factor		Retardation factor		Sleep disorder factor	

		Pretreatment	Posttreatment	Pretreatment	Posttreatment	Pretreatment	Posttreatment	Pretreatment	Posttreatment	Pretreatment	Posttreatment	Pretreatment	Posttreatment
The control group	60	29.2±6.7	12.0±7.4	10.3±3.4	3.8±2.7	1.2±1.0	0.5±0.7	3.4±2.4	4.0±3.9	8.0±1.7	2.8±1.5	6.3±3.5	2.2±1.6
The experimental group	60	28.3±6.4	9.1±7.0	10.1±3.7	2.4±2.4	1.4±1.2	0.3±0.4	2.7±2.0	2.6±2.5	7.8±1.8	2.2±1.3	6.4±2.2	1.5±1.1
*t*		0.88	2.14	2.29	2.78	1.16	1.78	1.561	2.66	0.15	2.36	0.17	2.58
*P*		0.38	0.04	0.82	0.01	0.25	0.08	0.12	0.01	0.89	0.02	0.87	0.01

The total score of the experimental group was decreased compared with that of the control group eight weeks after treatment, and the difference was statistically significant(Ρ<0.05) (Table-IV). Three cases of mild diarrhea were reported in the experimental group, which was improved after the dosages of SYD were reduced. Two cases of increased sweating, three cases of nausea, three cases of dry mouth, and one case of sexual dysfunction were reported in the control group, and all events were improved after the dosages of antidepressants were reduced.

**Table-IV:** Comparison of SSI between the two groups.

Groups	n	Pretreatment	Posttreatment
The control group	60	64.4±19.8	35.1±4.2
The experimental group	60	59.7±20.5	30.9±1.7
*t*		1.250	7.170
*P*		0.210	0.000

## DISCUSSION

The results of the present study showed no statistically significant difference in the total efficacy between the integrated Chinese and western medicine treatment and SSRI treatment alone, and significant differences, however, were observed in the total score and scores of factors of HAMD-17 between the two groups. Factors of HAMD-17 scale except body weight, including anxiety/somatic, cognitive impairment, retardation, and sleep disorders, were significantly improved in patients treated with SYD combined with SSRIs compared with those in patients treated with SSSRIs alone, indicating that integrated Chinese and Western medicine treatment can alleviate depressive symptoms in multiple dimensions, which may be explained by the advantages of multi-component, multi-target and overall regulatory effect of TCM. The efficacy and outcome of depression treatment are affected by various factors, and somatic symptoms are an important factor among others. A variety of somatic symptoms may occur in patients with depression in acute and remission phases, and some of these symptoms are difficult to locate or just subjective experience of physical discomfort with no organic lesions found after repeated examination. Studies have found that the somatic symptoms of depressive patients are positively correlated with the severity of depression^9^, and the improvement of somatic symptoms can be used to predict the development and prognosis of major depression. More severe somatic symptoms usually indicate increased suicidal tendencies, lower remission rate and worse quality of life.^10^ Previous studies have shown that antidepressants combined with TCM can alleviate somatic symptoms and make up for the deficiency of antidepressant monotherapy.^11^

The 5-HT hypothesis is a classic theory for the pathogenesis of depression. It is believed in this hypothesis that the decreased activity of the neurotransmitter 5-HT in the central nervous system is related to the depressive episodes. The 5-H system is involved in the regulation of sleep, eating, sexual behavior, pain, and circadian rhythms, and different degrees of impairments have been found in these processes in patients with depression. Based on this hypothesis, reuptake of 5-HT in the presynaptic membrane to increase the level of 5-HT in the synaptic cleft is the main pharmacological mechanism of SSRIs. SSRIs primarily include sertraline, escitalopram, paroxetine, and fluoxetine, which are class A antidepressants recommended by the Chinese Guidelines for the Prevention and Treatment of Depressive Disorders. These drugs, with few adverse reactions, are suitable for the treatment of patients with depression of all ages, and widely used in psychiatric hospitals.^12^

SYD, a classic prescription in “Treatise on the spleen and stomach”, astragalus membranaceus, Panax ginseng, and prepared licorice in the prescription can tonify the spleen and stomach, and nourish yang qi, and are the main components to treat the inhibitory somatic symptoms such as fatigue and weakness; wind-expelling components such as radix bupleuri, Saposhnikovia divaricate, notopterygium root, and Radix angelicae tuhuo can enhance Yang Qi, assist in the tonifying effect of Astragalus membranaceus and Panax ginseng, and also disperse Yin fire, which helps improve irritable symptoms; and Pinellia ternate and pericarpium citri reticulatae can eliminate dampness, and Coptis chinensis can clear dampness and heat. The whole prescription can regulate yin and yang, and ascend lucidity and descend turbidity, with both tonifying and dispersing effects.^13^

Modern pharmacological studies have found that TCMs with tonifying effects such as Astragalus membranaceus, Panax ginseng, and prepared licorice have antidepressant effects^14^, and the underlying mechanisms may involve the increased level of 5-HT in the hippocampus and striatum by Panax ginseng and Astragalus membranaceus, which can improve the synaptic structural abnormalities in the prefrontal cortex.^15^,^16^ Licorice effectively inhibits the apoptosis of hippocampal neurons.^17^ Prescriptions containing Radix bupleuri can upregulate the levels of 5-HT, norepinephrine(NE), and dopamine(DA) in the hippocampus of model mice^18^ to subsequently improve the depressive symptoms. Coptis chinensis can significantly improve the depression like behavior in rats, which probably be related to the antidepressant mechanism mediated by pro-inflammatory factors.^19^ Poria cocos and Pinellia ternata contain various effective TCM ingredients acting on antidepressant targets, with the favorable characteristics of multiple components, multiple targets, and multiple pathways in the treatment of depression.^20^

### Limitations:

It includes a small sample size, and interference from confounding factors such as research bias cannot be ruled out. Future multi-center trials are needed to obtain more extensive clinical evidence-based basis.

## CONCLUSIONS

SYD combined with SSRI antidepressants in the treatment of depression can reduce the total score and the scores of factors of HAMD-17, alleviate the symptoms of depression in multi-dimensions, and improve somatic symptoms without increasing the incidence of adverse drug reactions. This combination can be used as an integrated traditional Chinese and Western medicine treatment for depression.

### Authors’ Contributions:

**LL** and **YH:** Carried out the studies, participated in collecting data, and drafted the manuscript, and are responsible and accountable for the accuracy or integrity of the work.

**AW, MZ, LY** and **KM:** Literature search, performed the statistical analysis and participated in its design.

All authors have read and approved the final manuscript.
